# An analysis of the relationship of triglyceride glucose index with gastric cancer prognosis: A retrospective study

**DOI:** 10.1002/cam4.6837

**Published:** 2024-01-11

**Authors:** Chao Cai, Cheng Chen, Xiuli Lin, Huihui Zhang, Mingming Shi, Xiaolei Chen, Weisheng Chen, Didi Chen

**Affiliations:** ^1^ Department of Infectious Diseases The First Affiliated Hospital of Wenzhou Medical University, Hepatology Institute of Wenzhou Medical University, Wenzhou Key Laboratory of Hepatology Zhejiang China; ^2^ Wenzhou Medical University Wenzhou Zhejiang China; ^3^ Department of Gastrointestinal Surgery, National Key Clinical Specialty(General Surgery) The First Affiliated Hospital Of Wenzhou Medical University Zhejiang China; ^4^ Department of Hepatobiliary Surgery Affiliated Yueqing Hospital of Wenzhou Medical University; ^5^ Department of Radiation Oncology The First Affiliated Hospital, Wenzhou Medical University Zhejiang China

**Keywords:** gastric cancer, insulin resistance, prognosis prediction, triglyceride glucose index

## Abstract

**Aims/Introduction:**

Gastric cancer, one of the most common malignant tumors worldwide, is affected by insulin resistance. The triglyceride glucose (TYG) index is considered a surrogate indicator of insulin resistance; however, its prognostic value in patients with gastric cancer remains obscure. This study aimed to determine whether the TYG index could predict the long‐term prognosis of patients with gastric cancer after radical resection gastrectomy.

**Materials and Methods:**

We retrospectively analyzed patients with gastric cancer who underwent radical resection gastrectomy. The preoperative TYG index was calculated using the patients' laboratory data. Patients were divided into two groups based on a high or low TYG index. We observed overall survival and evaluated the clinical application value of the index using Cox proportional hazards regression to calculate independent parameters. A prediction model was also established.

**Results:**

In total, 822 patients with gastric cancer were included. The high and low TYG index groups comprised 353 and 469 patients, respectively. The overall survival time was significantly longer in the high‐index group than in the low‐index group. In the multivariate analysis, TYG index, preoperative age, surgical procedure, tumor node metastasis (TNM) stage, N stage, and postoperative complications (all *p* < 0.01) were considered independent prognostic predictors. Based on the multivariate analysis, the riglyceride glucose (TYG) index hazard ratio was 0.70 (95% confidence interval, 0.54–0.89, *p* = 0.004).

**Conclusions:**

We established a model with a high clinical application value and clinical practice relevance to predict the prognosis of gastric cancer. In this model, TYG was an independent protective factor for gastric cancer prognosis.

## 1 INTRODUCTION

Despite the recent decrease in the incidence of gastric cancer, it remains one of the most frequent malignancies,^1^ with most patients being diagnosed at a progressive or advanced stage. Gastric cancer, the fifth most common malignancy worldwide, has the third highest mortality rate, and is particularly prevalent in Southeast Asia (mainly China).^2^, ^3^ Radical surgical resection is still considered the only effective treatment,^4^ and postoperative recurrence and metastasis are the major causes of treatment failure.^5^ The 5‐year survival rate after radical gastrectomy for gastric cancer is less than 50%,^6^ and many factors influence its prognosis. Unlike early gastric cancer, middle‐ and late‐stage gastric cancers are associated with poor prognosis and high recurrence rates, and an accurate prognostic model for gastric cancer is lacking. Several predictive models exist for gastric cancer, including the ImmunoScore of Gastric Cancer (ISGC) classifier, which can effectively predict recurrence and survival and complement the prognostic value of the tumor node metastasis (TNM) staging system. The combination of ISGC and TNM staging has been shown to have a better prognostic value than TNM staging alone.^7^ Additionally, the Tumor‐infiltrating lymphocytes (TIL) model serves as a valuable diagnostic supplement because the TNM scoring system does not reflect the full range of information on the tumor microenvironment in gastric cancer. Specifically, high TIL levels are associated with a positive prognosis.^8^ Moreover, it should be acknowledged that most of these models predominantly rely on TNM staging and fail to comprehensively consider the various patient‐specific factors that may influence disease progression, including their nutritional status.^9^ However, an assessment of the nutritional status is crucial for achieving an all‐encompassing, individualized approach to prognostication in patients with gastric cancer.^10^, ^11^

The triglyceride glucose (TYG) index is a noninvasive surrogate indicator of insulin resistance^12^, ^13^, ^14^ that combines fasting plasma glucose and triglyceride levels. Furthermore, studies have revealed that the TYG index is a predictor for the development of multiple human diseases, including type 2 diabetes mellitus,^15^ cardiovascular diseases, and colorectal cancer.^16^, ^17^, ^18^ Several studies reported that insulin resistance is closely associated with gastric cancer prognosis.^19^, ^20^, ^21^ Martini et al.'s clinical research on prostate cancer demonstrated that the survival advantage of TYG might partly be attributed to the downregulation of certain oncogenes and/or the upregulation of programmed cell death protein 1 (PD‐1) expression determined by the immunosuppressive effect of obesity, ultimately leading to greater susceptibility to PD‐1 inhibitors.^22^ Okadome et al. speculated that TYG may affect esophageal cancer prognosis, such that patients' systemic nutritional and immunological status might affect their prognosis through local tumor immunity.^23^ However, few studies have examined the long‐term prognostic impact of TYG levels in patients with gastric cancer. In this study, we retrospectively investigated whether TYG expression is an independent prognostic factor for gastric cancer prognosis.

## 2 MATERIALS AND METHODS

### 2.1 Study population

We retrospectively analyzed the data of patients with gastric cancer who underwent curative resection at the First Affiliated Hospital of Wenzhou Medical University between July 2014 and March 2018. All clinical data were retrieved from the electronic medical records in the hospital database. The inclusion criteria were as follows: (a) radical resection gastrectomy, (b) pathological diagnosis of gastric carcinoma, and (c) biochemical blood examination performed less than 2 weeks prior to surgery. The exclusion criteria were as follows: (a) having been diagnosed with another malignant neoplasm or confirmed metastatic cancer; (b) having undergone other emergency operations during the 3 years before surgery; (c) having received preoperative chemotherapy or radiotherapy; and (d) inaccurate or incomplete medical records. The study protocol conformed to the ethical guidelines of the 1975 Declaration of Helsinki and was approved by the Review Committee of the First Affiliated Hospital of Wenzhou Medical University (2014063).

Routine clinical information retrieved from the electronic medical records included the following^1^: baseline characteristic information (e.g., age, sex, body mass index [BMI], and surgical history)^2^; blood parameters (e.g., fasting blood glucose, triglycerides, total cholesterol, plasma albumin)^3^; operative conditions (e.g., type of surgery, tumor location, tumor size, extent of lymph node dissection, TNM stage)^4^; postoperative conditions (e.g., postoperative complications (within 1 month of surgery), postoperative length of hospital stay, and long‐term survival time).

The tumor stage was classified in accordance with the American Joint Commission on Cancer 8th edition guidelines.^24^ Postoperative complications were defined as greater than class II according to the Clavien–Dindo classification.^25^ All quality assessments and risk of bias evaluations were independently completed by two researchers who were blinded to other data.

### 2.2 TYG evaluation

The TYG index was calculated using the following formula: TYG index = ln [(fasting triglycerides) × fasting glucose/2] (mg/dL). We selected the critical point of the preoperative TYG using the enumeration method in the X‐tile program (X‐Tile software version 3.6.1, Yale University), which is the value with the maximal Youden index. Thus, according to the cutoff point, all patients were divided into high‐ and low‐TYG index groups.

### 2.3 Statistical analysis

For the distribution of continuous data, the Kolmogorov–Smirnov test was applied to verify normality before hypothesis testing. Continuous variables are expressed as mean value ± standard deviation of the mean (SD), whereas non‐normally distributed.

Data are presented as medians and interquartile ranges. Fisher's exact test, *χ* ^2^‐test, and *t*‐test were used to compare baseline characteristics between the groups. Overall survival (OS) curves were constructed using the Kaplan–Meier method.

Analyses were performed using the log‐rank test. Cox regression was performed and proportional hazards model was established. Hazard ratios (HRs) and 95% confidence intervals (CIs) were calculated. Differences were considered statistically significant at *p* < 0.05. All statistical analyses were performed using IBM SPSS Statistics for Windows, version 25.0, (IBM Corp., Armonk, NY, USA) and R statistical programming software, version 3.6.3 (The R Foundation, Vienna, Austria).

### 2.4 Development of the prognosis prediction model

We developed a prognosis prediction model, illustrated using a nomogram chart, calculated the C‐index, and performed a curve analysis. The above analyses were performed using the R statistical programming software (version 3.6.3).

## 3 RESULTS

**FIGURE 1:**
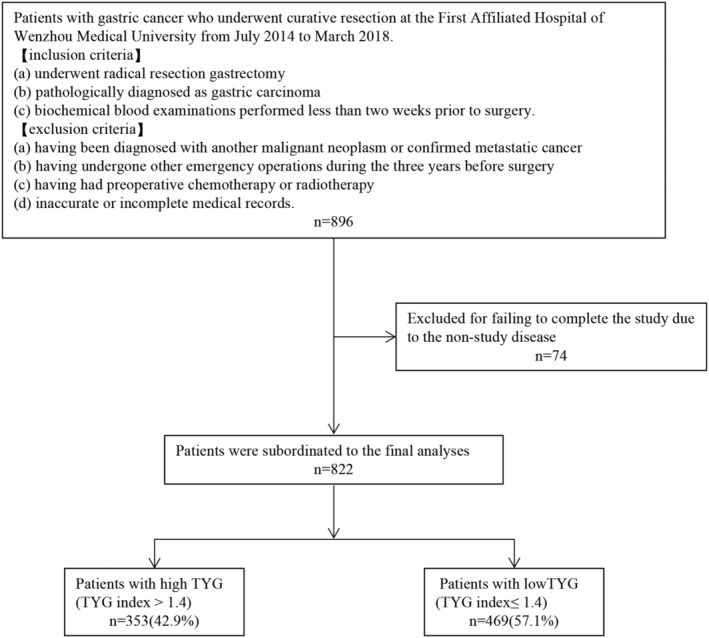
Flow diagram of the eligibility and exclusion criteria of the current study. TYG, triglyceride glucose index.

We retrospectively assessed 896 patients with gastric cancer between July 2014 and March 2018 for inclusion in this study and finally included 822 (Figure 1 shows the flowchart of the selection and screening of patients). Based on a cutoff point of 1.4 for the TYG index, patients were assigned to one of two groups: high (>1.4) and low (≤1.4) TYG index. The high and low index groups comprised 353 (42.9%) and 469 (57.1%) patients, respectively.

### 3.1 Patient characteristics

Of the 822 patients enrolled in this study, 73.2% were men, and 26.8% were women. The mean (SD) TYG index was 1.37 (±0.67). Table 1 presents the clinical and demographic characteristics of the two groups. There were significant differences between the two groups in BMI, TYG, fasting glucose, fasting triglycerides, Charlson score, preoperative diabetes mellitus, and hypertension (all *p* < 0.001).

**TABLE 1:** Preoperative backgrounds and comparison of backgrounds based on triglyceride glucose index (data are shown in mean ± SD).

Variables	Overall Mean ± SD (%) [*n* = 822]	High TYG Mean ± SD (%) [*n* = 353]	Low TYG index Mean ± SD (%) [*n* = 469]	*p*‐Value
Age, y	64.65 ± 10.81	64.68 ± 9.88	64.64 ± 11.48	0.956
Gender				0.067
Male	602 (73.2)	247 (70.0)	355 (75.7)	
Female	220 (26.8)	106 (30.0)	114 (24.3)	
BMI, kg/m^2^	24.54 ± 9.58	23.52 ± 2.85	21.81 ± 3.00	^a^<0.001
TYG	1.37 ± 0.67	1.98 ± 0.51	0.92 ± 0.32	^a^<0.001
Fasting Glucose	6.42 ± 2.54	7.63 ± 3.00	5.52 ± 1.35	^a^<0.001
Fasting Triglycerides	1.54 ± 1.14	2.72 ± 1.41	0.98 ± 0.31	^a^<0.001
ASA				0.124
1–2	709 (86.3)	312 (88.4)	397 (84.6)	
≥ 3	113(13.7)	41(11.6)	72(15.4)	
Charlson score				^a^<0.001
0	491(59.7)	172(48.7)	319(68.0)	
1–2	293(35.6)	158(44.8)	135(28.8)	
3–6	38(4.6)	23 (6.5)	15 (3.2)	
NRS				^a^<0.01
1–2	519 (63.1)	246 (69.7)	273 (58.2)	
3–4	221 (26.8)	84 (23.8)	137 (29.2)	
5–6	82 (10.0)	23 (6.5)	59 (12.6)	
Surgical history				0.289
No	659 (80.2)	289 (81.9)	370 (78.9)	
Yes	163 (19.8)	64 (18.1)	99 (21.1)	
Abdominal surgery history				0.596
No	716 (87.1)	310 (87.8)	406 (86.6)	
Yes	106 (12.9)	43 (12.2)	53 (13.4)	
Preoperative diabetes				*<0.001
No	726 (88.3)	283 (80.2)	443 (94.5)	
Yes	96 (11.7)	70 (19.8)	26 (5.5)	
Hypertension				^a^<0.001
No	594 (72.3)	227 (64.3)	367 (78.3)	
Yes	228 (27.7)	126 (35.7)	102 (21.7)	
Laparoscopic surgery				0.502
No	599 (72.9)	253 (71.7)	346 (73.8)	
Yes	223 (27.1)	100 (28.3)	123 (26.2)	
Surgical procedure				0.513
SG	525 (63.9)	221 (62.6)	304 (64.8)	
TG	297 (36.1)	132 (37.4)	165 (35.2)	
Type of reconstruction				0.340
B‐I	355 (43.2)	153 (43.3)	202 (43.1)	
B‐II	126 (15.3)	47 (13.3)	79 (16.8)	
Roux‐en‐Y	341 (41.5)	153 (43.3)	188 (40.1)	
Combined resection				0.281
No	749 (91.1)	326 (92.4)	423 (90.2)	
Yes	73 (8.9)	27 (7.6)	46 (9.8)	
Surgical durations (min)	202.11 ± 53.41	200.96 ± 52.19	202.96 ± 54.34	0.614
Histologic type				0.906
Undifferentiated	424 (51.6)	183 (51.8)	241 (51.4)	
Differentiated	398 (48.4)	170 (48.2)	228 (48.6)	
Tumor site				0.225
Upper	117 (14.2)	60 (17.0)	57 (12.2)	
Middle	229 (27.9)	91 (25.8)	138 (29.4)	
Low	449 (54.6)	191 (54.1)	258 (55.0)	
Mixed	27 (3.3)	11 (3.1)	16 (3.4)	
T stage				^a^ 0.018
1–2	314 (38.2)	155 (43.9)	159 (33.9)	
3–4	508 (61.8)	198 (56.1)	310 (66.1)	
N stage				0.227
0	370 (45.0)	171 (48.4)	199 (42.4)	
1	140 (17.0)	61 (17.3)	79 (16.8)	
2	151 (18.4)	58 (16.4)	93 (19.8)	
3	161 (19.6)	63 (17.8)	98 (20.9)	
TNM stage				^a^ 0.026
I	267 (32.5)	132 (37.4)	135 (28.8)	
II	179 (21.8)	75 (21.2)	104 (22.2)	
III	376 (45.7)	146 (41.4)	230 (49.0)	
Postoperative complications				0.772
NO	605 (73.6)	258 (73.1)	347 (74.0)	
YES	217 (26.4)	95 (26.9)	122(469)	

Abbreviations: ASA, American Society of anesthesiologists; BMI, body mass index; NRS, nutritional risk screening; TYG, triglyceride glucose index; SD: standard deviation; SG, subtotal gastrectomy; TG, total gastrectomy.

^a^ Statistically significant (*p* < 0.05).

### 3.2 Clinicopathologic characteristics of patients with gastric cancer

TYG was significantly correlated with age, BMI, American Society of Anesthesiologists, nutritional risk screening (NRS), laparoscopic surgery, surgical procedure, combined resection, TNM stage, histologic type, and postoperative complications. No significant differences were observed in other clinicopathological characteristics. Univariate analyses indicated that age, NRS, TYG, hemoglobin (HB), albumin (ALB), tumor site, laparoscopic surgery, surgical procedure, type of reconstruction, combined resection, T stage, N stage, TNM stage, histologic type, and postoperative complications (all *p* < 0.001) differed significantly according to prognosis (Table 2).

**TABLE 2:** Prognostic factors for overall survival (data are shown in mean ± SD).

Variables	Univariate analysis		Multivariate analysis	
	HR (95% CI)	*p* Value	HR (95% CI)	*p* Value
TYG (low/high)	0.66(0.51–0.84)	0.001	0.70(0.54–0.89)	^a^ 0.004
Gender	1.32(1.00–1.75)	0.053	–	–
Age (≧70/﹤70)	1.84(1.46–2.33)	<0.001	1.59(1.25–2.03)	^a^ <0.001
BMI (≧25/﹤25)	0.69(0.49–0.96)	0.025	–	–
ASA	1.45(1.07–1.96)	0.016	–	–
NRS	1.64(1.30–2.08)	<0.001	–	–
Charlson score	1.17(1.01–1.37)	0.040	–	–
Previous surgery	0.99(0.74–1.33)	0.936	–	–
Previous abdominal surgery	0.95(0.67–1.36)	0.790	–	–
HB (≧100 g/L/﹤100 g/L)	1.64(1.30–2.07)	<0.001	–	–
ALB(≧35/﹤35 g/L)	1.78(1.38–2.28)	<0.001	–	–
Diabetes mellitus	1.05(1.73–1.51)	0.785	‐	‐
Tumor site				
Upper	1.12(0.79–1.60)	0.532	‐	‐
Middle	1.22(0.93–1.60)	0.143	‐	–
Low	1	0.002	–	–
Mixed	2.73(1.62–4.58)	<0.001	–	–
Laparoscopic surgery	0.52(0.38–0.71)	<0.001	–	–
Surgical procedure (SG/TG)	1.92(1.52–2.43)	<0.001	1.46(1.15–1.86)	^a^ 0.002
Combined resection	1.91(1.34–2.71)	<0.001	–	–
Type of reconstruction				
B–I	1	<0.001	–	–
B–II	1.92(1.36–2.72)	<0.001	–	–
Roux‐en‐Y	2.26(1.72–2.96)	<0.001	–	–
Surgical durations	1.001(1.000–1.003)	0.116	–	–
T stage	1.68(1.49–1.88)	<0.001	–	–
N stage	1.78(1.62–1.97)	<0.001	1.37(1.17–1.60)	^a^<0.001
TNM stage				
I	1	<0.001	1	^a^<0.001
II	2.85(1.80–4.50)	<0.001	1.88(1.17–3.02)	^a^ 0.010
III	7.09(4.80–10.47)	<0.001	3.04(1.80–5.13)	^a^<0.001
Histologic type				
High differentiated	1	<0.001	–	–
Moderately differentiated	2.54(1.50–4.32)	0.001	–	–
Undifferentiated	3.41(2.04–5.70)	<0.001	–	–
Postoperative complications	2.17(1.71–2.76)	<0.001	1.79(1.39–2.29)	^a^<0.001

Abbreviations: ALB, albumin; ASA, American Society of anesthesiologists; BMI, body mass index; CI, confidence interval; HB, hemoglobin; HR, hazard ratio; NRS, nutritional risk screening; SG, subtotal gastrectomy; TG, total gastrectomy; TYG, triglyceride glucose index.

^a^ Statistically significant (*p* < 0.05).

Fourteen variables that were statistically significant in univariate regression analyses were selected for multivariate analyses as potential independent factors. Based on multivariate analysis, five of the fourteen variables were independent factors for prognosis prediction (*p* < 0.05): TYG (HR, 0.70; 95% CI, 0.54–0.89; *p* = 0.004); age (HR, 1.59; 95% CI, 1.25–2.0; *p* < 0.001); surgical procedure (HR, 1.46; 95% CI, 1.15–1.86; *p* = 0.002); N stage (HR,1.37; 95% CI, 1.17–1.60; *p* < 0.001); TNM stage (II vs. I: HR, 1.88, 95% CI,1.17–3.02; p = 0.001); (III vs. I: HR,3.04; 95% CI,1.80–5.13; *p* < 0.001)] and postoperative complications (HR,1.79; 95% CI,1.39–2.29; *p* < 0.001).

The Kaplan–Meier curves of OS for the high and low TYG index groups are shown in Figure 1. Patients in the low TYG index group had a poorer prognosis than did those in the high TYG index group (*p* < 0.001).

**FIGURE 2:**
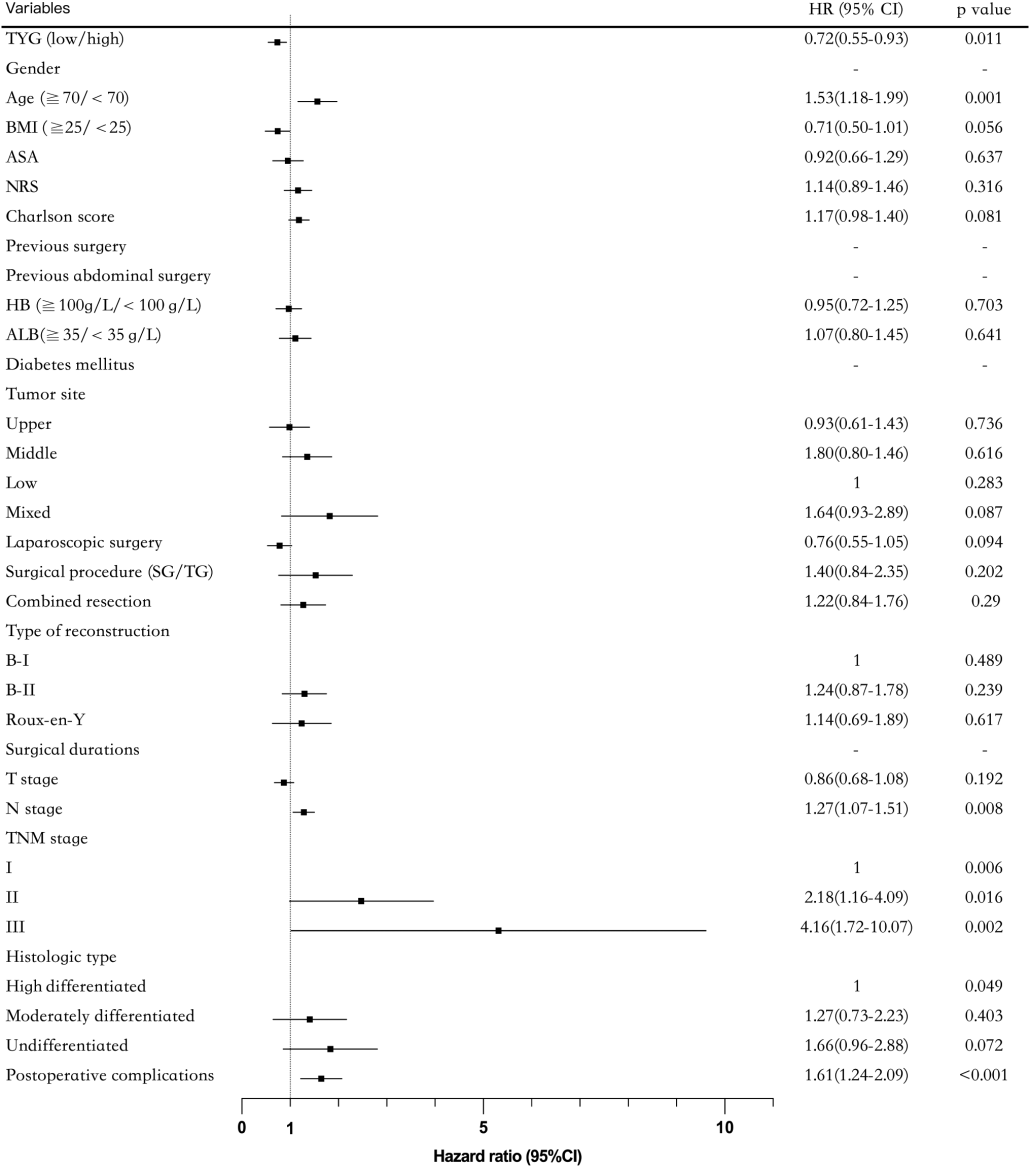
Forest plot showing the results of multivariate Cox regression analysis and visualizing the hazard ratios of the clinicopathologic characteristics for gastric cancer prognosis. ALB, albumin; ASA, American Society of anesthesiologists; BMI, body mass index; HB, hemoglobin; NRS, nutritional risk screening; SG, subtotal gastrectomy; TG, total gastrectomy; TYG, triglyceride glucose index.

A nomogram for predicting the probability of 1‐ and 3‐year survival is shown in Figure 2.

**FIGURE 3:**
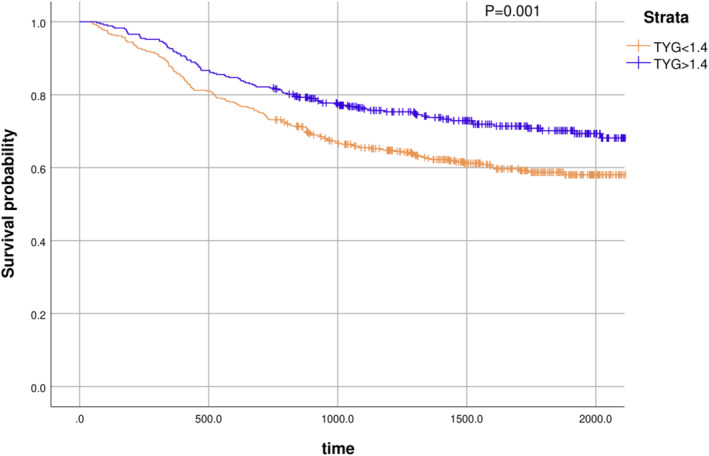
Kaplan–Meier survival curve analyses for overall survival among 822 patients who underwent radical resection gastrectomy. TYG, triglyceride glucose index.

**FIGURE 4:**
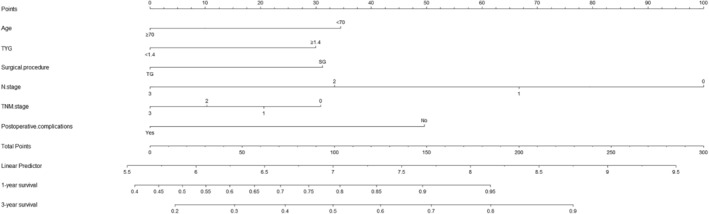
A nomogram indicating the survival. An example of a nomogram—Draw an upward vertical line from the covariate to the points bar to calculate points. Based on the sum of the covariate points, draw a downward vertical line from the total points line to calculate survival rate. TYG, triglyceride glucose index; TNM stage, tumor node metastasis stage.

**FIGURE 5:**
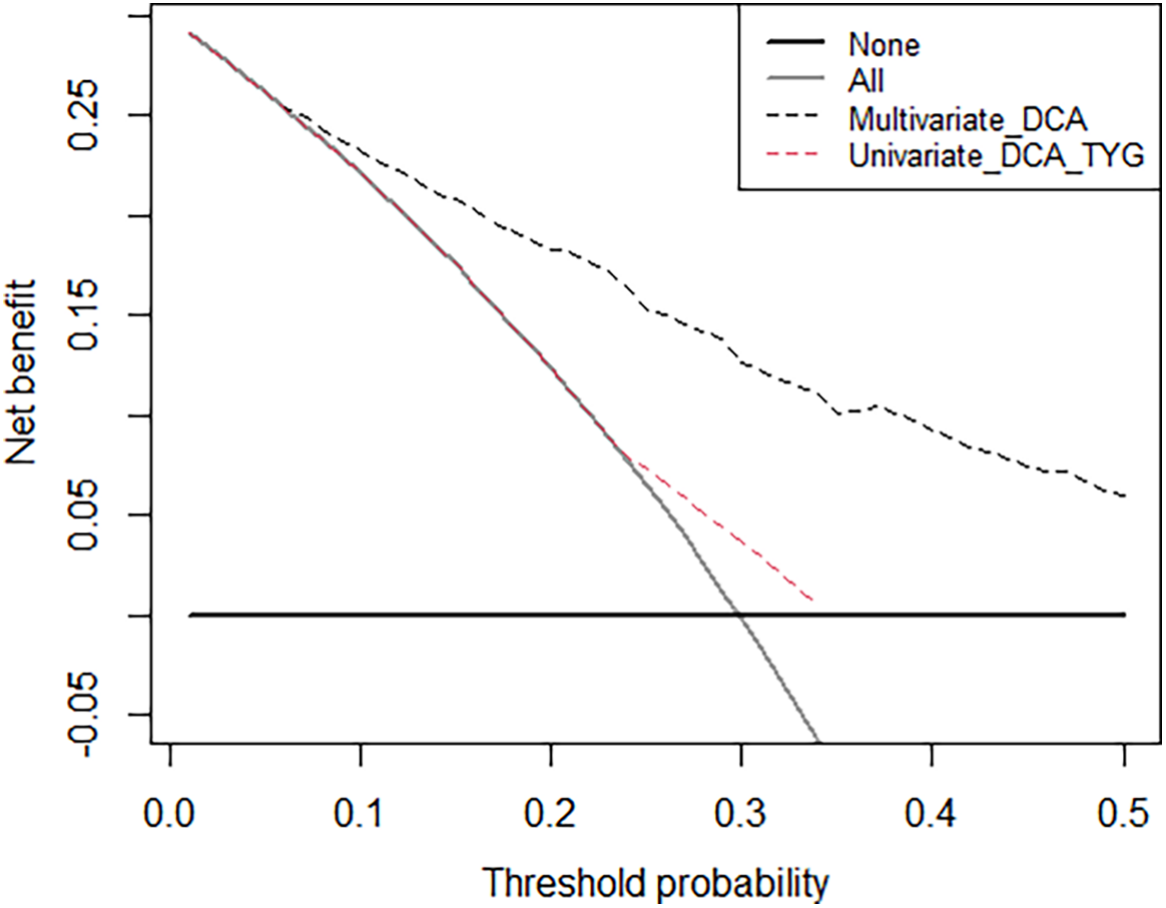
Decision curve analysis for prediction model with TYG. DCA, decision curve analysis; TYG, triglyceride glucose index.

Decision curve analysis was used to assess the net benefits for a range of threshold probabilities of the predictive model (Figure 3), and the clinical utility of TYG was evaluated (Figure 4 and 5).

## 4 DISCUSSION

Patients diagnosed with advanced gastric cancer have a high mortality rate, poor prognosis following surgery, and a low survival rate.^26^ However, the predictive ability of existing research and prognostic prediction models is generally limited and cannot fully reflect the patients' disease status, owing to the absence of nutritional status assessment.^9^, ^10^, ^11^ Notably, TYG appears to be associated with the nutritional status of patients. Moreover, a previous study found that TYG is a risk factor for the development of gastric cancer in a health checkup cohort^27^; however, research on the effect of TYG on the prognosis of patients with gastric cancer who have undergone surgery is lacking. In our study, we retrospectively evaluated 822 patients whose findings showed that the prognosis of patients after radical gastric cancer surgery was related to age, NRS, TYG, HB, ALB, tumor site, laparoscopic surgery, surgical procedure, type of reconstruction, combined resection, T stage, N stage, TNM stage, histologic type, and postoperative complications. The multivariate analysis revealed that age, TYG, N stage, TNM stage, surgical approach, and postoperative complications significantly affected the prognosis of radical gastric cancer surgery. Thus, we established a prognostic prediction model based on the findings of this retrospective clinical data analysis. In this model, TYG was an independent protective factor for gastric cancer prognosis. Specifically, a higher TYG level indicated a better prognosis and patients' nutritional status. Finally, decision curve analysis revealed that the model had good clinical guidance value.

It is generally accepted that TNM staging can objectively and accurately predict the prognosis of gastric cancer.^28^ Published by the International Union against Cancer (IUCC), TNM staging is a globally accepted method for classifying tumors. It captures the tumor's biological behavior and rate of disease progression through T, N, and M staging, representing the depth of tumor infiltration, lymph node metastasis, and distant metastasis, respectively. In our study, TNM stage data were analyzed in accordance with the 8th edition of the IUCC/AJCC (TNM) system. The results of the multivariate analysis indicated that TNM staging is an independent risk factor for gastric cancer,^29^ which is consistent with research reports from both domestic and international sources.^29^, ^30^ Thus, TNM staging provides a scientific foundation for predicting the prognosis of gastric cancer.

A retrospective clinical data analysis revealed that a prediction model consisting of TYG (HR = 0.7) and TNM stage (II, HR = 1.88; III, HR = 3.04) could accurately predict the prognosis of patients with gastric cancer. Similarly, decision curve analysis proved that the combined model has a high clinical application value and accurate predicting capabilities. Further, survival analysis revealed that the high TYG group had a higher survival rate than did the low TYG group (*p* < 0.05). These results suggest that TYG is an independent protective factor for the prognosis of patients with gastric cancer who undergo radical surgery. The TYG index, a surrogate marker of insulin resistance, correlates well with the gold standard hyperglycemic clamp and can be used in epidemiological surveys to assess the prevalence of diabetes and insulin resistance.^12^, ^13^, ^14^ Among the 822 patients in our study, the independent risk factor analysis results for the presence or absence of diabetes were not statistically significant. This implies that the TYG index is a more accurate predictor of gastric cancer prognosis than diabetes history and may reflect the relationship between human energy metabolism and tumor prognosis.

Our study found that TYG was a protective factor for the survival of patients with gastric cancer after surgery, which is not entirely consistent with the results of a previous study.^27^ Our analysis suggests that in patients diagnosed with postoperative gastric cancer, the impact of TYG on their postoperative prognosis may be due to the following reasons. First, TYG can reflect lipid metabolism, and the survival advantage of high TYG might be partly attributed to the downregulation of certain oncogenes and/or upregulation of PD‐1 expression, as determined by the immunosuppressive effect of obesity, ultimately leading to greater susceptibility to PD‐1 inhibitors.^22^ Second, TYG can reflect glucose metabolism, and the glycolytic pathway is a potential target for controlling inflammation. Notably, activated immune cells depend on glycolytic metabolism to fuel rapid ATP production and provide biosynthetic materials for growth and proliferation. Activated immune cells require a large influx of glucose to fuel glycolysis.^31^ Furthermore, TYG reflects patients' nutritional status and affects their immune status. Patients' systemic nutritional and immunological status may affect their prognosis through local tumor immunity.^23^ Previous studies in healthy individuals have suggested that TYG affects the occurrence and development of gastric cancer via lipotoxicity. Increased adipose tissue mass and dysfunctional adipose tissue induce systemic lipid overflow and inflammation by altering adipokine and cytokine secretion. High triglyceride levels are also associated with the severity and progression of malignancy.^27^ However, this is not the main factor affecting the postoperative prognosis of patients with gastric cancer. Thus, it is crucial to maintain a healthy life before and after tumor development to improve the prognosis with proper nutritional intake.

In our study, TYG was found to be an independent protective factor for the prognosis of patients with gastric cancer, which differs from the findings of previous studies. Combining recent related research with our results, we propose that tumor cells obtain their nutrients and energy primarily through aerobic glycolysis^32^ and that a high TYG index indicates insulin resistance. Specifically, a higher TYG index indicates a poorer ability to utilize glucose, resulting in tumor cell growth restriction. Blood lipid levels reflect the patients' nutritional status and lower blood lipid levels may indicate cachexia or malnutrition in the patient,^33^ which may also lead to a worse prognosis. Notably, for postoperative patients, especially those who have undergone gastrointestinal tumor surgery, the overall nutritional status might be crucial in influencing survival.

## 5 LIMITATIONS

To our knowledge, this is the first retrospective study to analyze whether the TGY index could predict the long‐term prognosis of patients with gastric cancer after radical resection gastrectomy. This finding is significant as it provides a reference point. Inevitably, this study presents some limitations. First, this was a retrospective study, and unavoidable selection bias may be present. Second, this study lacked a validation cohort. In addition, the findings still require further validation through prospective clinical trials or in vivo and in vitro experiments. Nevertheless, our research is the first to show that the TYG index can be a protective factor for postoperative gastric cancer; furthermore, we provide important and effective models for predicting survival in patients with gastric cancer.

## 6 CONCLUSION

In summary, we found that age, TYG index, N stage, TNM stage, surgical procedure, and postoperative complications are prognostic factors for gastric cancer. Based on these factors, we established a model to predict the prognosis of gastric cancer, with high clinical application value and better clinical practice ability.

In this model, TYG was an independent protective factor for gastric cancer prognosis. A higher TYG index indicated a better prognosis. This is the first retrospective study to show that TYG index can be used as a protective factor for postoperative gastric cancer, providing a new noninvasive protective indicator for survival prediction in patients with tumors. More importantly, our findings may guide us to provide better and more reasonable support for patients with tumors in clinical practice in the future. Thus, the relationship between TYG index and prognosis in patients without diabetes but with gastric cancer before surgery should be further examined.

## AUTHOR CONTRIBUTIONS

**Chao Cai:** Data curation (equal); formal analysis (equal); writing – original draft (equal); writing – review and editing (equal). **Cheng Chen:** Data curation (equal); formal analysis (equal); writing – original draft (equal); writing – review and editing (equal). **Xiuli Lin:** Data curation (supporting); writing – review and editing (supporting). **Huihui Zhang:** Writing – review and editing (supporting). **mingming shi:** Writing – review and editing (supporting). **Ji Lin:** Writing – review and editing (supporting). **Xiaolei Chen:** Writing – review and editing (supporting). **Weisheng Chen:** Conceptualization (supporting); funding acquisition (supporting); writing – review and editing (supporting). **Didi Chen:** Conceptualization (lead); funding acquisition (lead); writing – review and editing (supporting).

## FUNDING INFORMATION

National Key Clinical Specialty (General Surgery), The First Affiliated Hospital Of Wenzhou Medical University. Wenzhou Science and Technology Plan Project: Y2020123; Y2023111. Zhejiang Medical and Health Science and Technology Program: 2024KY139. General Project of Education Department of Zhejiang Province: Y202353447.

## CONFLICT OF INTEREST STATEMENT

The Chao Cai, Cheng Chen, Xiuli Lin, Huihui Zhang, Mingming Shi, Ji Lin, Xiaolei Chen, Weisheng Chen and Didi Chen declare that the research: An analysis of the relationship of triglyceride glucose index with gastric cancer prognosis: a retrospective study was conducted in the absence of any commercial or financial relationships that could be constructed as a potential conflict of interest.

## ETHICS STATEMENT

The study protocol conformed to the ethical guidelines of the 1975 Declaration of Helsinki and was approved by the review committee of the First Affiliated Hospital of Wenzhou Medical University (2014063).

## Data Availability

The data used to support the findings of this study are available from the corresponding author upon request.
